# Interactions between extracorporeal support and the cardiopulmonary system

**DOI:** 10.3389/fphys.2023.1231016

**Published:** 2023-09-12

**Authors:** Kaspar F. Bachmann, David Berger, Per Werner Moller

**Affiliations:** ^1^ Department of Intensive Care Medicine, Inselspital, Bern University Hospital, University of Bern, Bern, Switzerland; ^2^ Department of Anaesthesiology and Intensive Care, University of Tartu, Tartu, Estonia; ^3^ Department of Anaesthesia, SV Hospital Group, Institute of Clinical Sciences at the Sahlgrenska Academy, University of Gothenburg, Gothenburg, Sweden

**Keywords:** ECMO, gas exchange, venous return, cardiac output, pulmonary physiology, membrane lung

## Abstract

This review describes the intricate physiological interactions involved in the application of extracorporeal therapy, with specific focus on cardiopulmonary relationships. Extracorporeal therapy significantly influences cardiovascular and pulmonary physiology, highlighting the necessity for clinicians to understand these interactions for improved patient care. Veno-arterial extracorporeal membrane oxygenation (veno-arterial ECMO) unloads the right ventricle and increases left ventricular (LV) afterload, potentially exacerbating LV failure and pulmonary edema. Veno-venous (VV) ECMO presents different challenges, where optimal device and ventilator settings remain unknown. Influences on right heart function and native gas exchange as well as end-expiratory lung volumes are important concepts that should be incorporated into daily practice. Future studies should not be limited to large clinical trials focused on mortality but rather address physiological questions to advance the understanding of extracorporeal therapies. This includes exploring optimal device and ventilator settings in VV ECMO, standardizing cardiopulmonary function monitoring strategies, and developing better strategies for device management throughout their use. In this regard, small human or animal studies and computational physiological modeling may contribute valuable insights into optimizing the management of extracorporeal therapies.

## Introduction

Extracorporeal support is increasingly used as a rescue strategy in cardiogenic shock, including cardiac arrest (eCPR), where veno-arterial (VA) extracorporeal membrane oxygenation (ECMO) can support the heart and lung functions temporarily (Abrams et al., 2022), or in acute respiratory failure where veno-venous (VV) ECMO is used to support lung function (Combes et al., 2022). During the last two decades, the use of both ECMO modalities has increased almost exponentially, with over 151.000 treatments registered in the ELSO registry through 2020 (Vyas and Bishop, 2022).

During VA ECMO, venous blood is usually drained from the right atrium and/or caval veins and returned to the arterial system after oxygenation and decarboxylation in the oxygenator (Jayaraman et al., 2017). The return cannula can be situated centrally in the ascending aorta, as is common for post-cardiotomy patients, or percutaneously in the femoral artery, as is done in acute cardiogenic shock or during eCPR (Jayaraman et al., 2017). In VV ECMO, venous blood is usually drained from the IVC and returned to the right atrium directly, or via injection in the superior vena cava (SVC). The right heart then pumps already oxygenated and decarboxylated blood through the lung, to the left heart which in turn pumps the blood into the arterial circulation (Jayaraman et al., 2017). Since VV ECMO requires adequate right and left ventricular function, it is primarily used for patients with severe pulmonary failure.

Different ECMO modalities and their associated cannulation techniques have significantly different physiological impact on cardiopulmonary physiology. VV ECMO mainly interacts with the right heart and pulmonary function as it may decrease right ventricular afterload (Petit et al., 2021), whereas VA ECMO bypasses - and affects - the entire cardiopulmonary unit and its function.

The purpose of this review is to describe these interactions for different ECMO modalities, and to illustrate physiological concepts that are relevant to properly manage extracorporeal support for severe cardiac and/or pulmonary failure. Table 1 lists all the interactions presented in this review and explains the underlying physiological concepts, the clinical consequences and possible management strategies.

**TABLE 1:** Interactions between extracorporeal support and the cardiopulmonary system.

Native system	Artificial system	Physiological interaction	Clinical impact	Management
Cardiovascular	VA ECMO	Increase in LV afterload	LV distension and reduced function, pulmonary edema	Unloading of LV, cannulation strategy
Cardiovascular	VA ECMO	Venous return function	Flow limitation during VA ECMO	Increase in MSFP or reduction in R_VR_
Cardiovascular	VA ECMO	Opposing flow from two circuits	Differential hypoxia (Harlequin, North-South)	Cannulation strategy
Pulmonary	VA ECMO	Transfer of gas exchange	Monitoring of limited lung function	Weaning strategy
Pulmonary	VA ECMO	Flow cessation through lung	Lung deterioration	Partial unloading, pneumonia treatment, bronchoscopy
Cardiovascular	VV ECMO	Reduced RV strain	Reduced RV failure	Optimize device and ventilator settings with regards to RV function
Cardiovascular	VV ECMO	Venous admixture and oxygen saturations	Curvilinear function of arterial saturation	Management of high and low cardiac output
Pulmonary	VV ECMO	CO_2_ elimination through artificial lung	Limiting ventilator induced lung injury	Ventilator strategies
Pulmonary	VV ECMO	Changes in respiratory quotient and nitrogen content	Changes in gas exchange and end-expiratory lung volumes	Management of ECMO sweep gas flow oxygen fraction

## Veno-arterial ECMO

### Interaction with left ventricular afterload

In VA ECMO, oxygenated and decarboxylated blood is returned to the arterial system (Jayaraman et al., 2017). Since the left ventricle (LV) must eject against an increased aortic pressure caused by the ECMO inflow, this inherently increases LV afterload. Depending on the LV state, this may have severe negative effects (Burkhoff et al., 2015). In a pressure-volume plot, this is seen as a rightward-upward shift of the loop, with an increase in aortic elastance (E_a_) along with an increase in LV filling volume and end-diastolic pressure (Burkhoff et al., 2015). Because the LV end-diastolic pressure increases exponentially at higher filling volumes, this may be most relevant for an already distended and impaired left ventricle, as is frequently seen in cardiogenic shock (Burkhoff et al., 2015). These effects lead to reduced LV ejection fraction and decreased stroke volume (Truby et al., 2017), and can promote pulmonary edema and cardiac ischemia, and may increase the risk for LV thrombus - all factors ultimately impairing myocardial and patient recovery (Grandin et al., 2022). Right heart function further determines the extent of LV overload, where an increased right ventricular function, e.g., increased end-systolic pressure volume relationship (ESPVR) may overload a dysfunctional LV further (Donker et al., 2021). Strategies to reduce LV afterload and the accompanying distension include either pharmacological afterload reduction, primarily through the use of vasodilators, or blood volume reduction, or if necessary mechanical unloading by means of an Impella device, intra-aortic balloon pump or LV venting catheters (Burkhoff et al., 2015; Donker et al., 2019; Kowalewski et al., 2020). Recent studies and meta analyses show that between 26.7% and 49.0% of patients undergoing VA ECMO receive mechanical unloading and mechanical unloading was associated with a decreased mortality (Kowalewski et al., 2020; Schrage et al., 2020; Grandin et al., 2022), but data from prospective trials are missing. Mechanical unloading has been associated with an increased VA ECMO weaning success (Kowalewski et al., 2020), but a higher complication rate has been observed (Schrage et al., 2020; Grandin et al., 2022). Entry and exit strategies for mechanical unloading in VA ECMO will need further clarification in future research and management strategies for LV overload should be tailored to the underlying pathophysiological process (Donker et al., 2022). If mechanical unloading is applied, an early strategy may be beneficial (Schrage et al., 2023).

### Venous return as a limiting factor in extracorporeal support

Guyton’s model of venous return (VR) has been the subject of debate. Using a series of carefully designed physiological experiments (Berger et al., 2016a; Moller et al., 2017; Moller et al., 2019) we have addressed arguments previously raised against the model (Werner-Moller et al., 2020). Although the full nuance of the debate is beyond the scope of this review, the following reasoning rests unaffected by any remaining controversies. Since the blood volume exceeds the unstressed capacitance, the circuit is pressurized by a stressing volume which creates a positive transmural vascular pressure, present also at zero flow. This is a manifestation of the static energy stored in the vessel walls (Magder, 2016). Mean systemic filling pressure (MSFP) is the equilibrated arteriovenous pressure at zero flow and equals the ratio of stressed vascular volume to the systemic vascular compliance (Werner-Moller et al., 2022). During flow, ventricular and/or ECMO pump work shifts part of this stressing volume from the venous to the arterial compartment. A prerequisite for flow is the presence of a pressure gradient pushing blood from points of higher pressure to points of lower pressure. Venous return requires MSFP to exceed right atrial (RA) pressure (P_RA_) and the flow is opposed by the resistive properties of (mainly, but not exclusively) venous vessels (resistance to VR: R_VR_) (Berger et al., 2016b; Berger et al., 2019). While acknowledging that R_VR_ is a mathematical abstraction representing complex physiology, it nevertheless can be expressed with the following relationship:

$$Venous\;return=\frac{MSFP-P_{RA}}{R_{VR}}$$

The soft vessel walls of the vascular circuit will start to collapse as the distending transmural pressure decreases to zero (Moller et al., 2017; Moller et al., 2019). When right-sided pump function (the native RV or an ECMO pump) drains more volume than the VR return function can push *from the periphery into the RA*, vessels collapse, further limiting flow as resistance increases towards infinity. In this situation, cardiac output and/or ECMO flow cannot be increased by increases in pump speed unless VR be increased firstly. According to the above presented formula, this can only be achieved by increasing stressed volume and MSPF, by means of vasopressors and/or volume expansion, or decreasing the resistance to venous return (R_VR_) (Moller et al., 2019).

### Differential hypoxia due to differing oxygen content in the native and artificial arterial system

In the setting of VA ECMO, the native arterial and artificial flows with differing oxygen and carbon dioxide contents, both enter the arterial system (Berger et al., 2023). In addition to an increase in left ventricular afterload, oxygenated blood from the artificial circuit may not reach all organs depending on cannulation strategy and left ventricular function (Wilson et al., 2022). In patients treated with peripherally cannulated VA ECMO with an improving left ventricular function but poor pulmonary function, differential hypoxia (a.k.a. north-south or harlequin phenomenon) may become a concern: Poorly oxygenated blood from the LV reaches the right and upper quadrants of the body, including heart and brain, while the highly oxygenated blood from the membrane lung injected into the femoral artery cannot reach these important organs (Blandino Ortiz et al., 2021). Monitoring of differential hypoxia may be done comparing blood gas analyses from the right radial artery against blood drawn from the left radial or femoral artery. Differential hypoxia may also be present in the venous system during VA ECMO: oxygen saturations in the blood drained from the IVC may differ substantially from the oxygen saturations in the pulmonary artery (Berger et al., 2023) and SVC (Hou et al., 2015). Progressively decreasing pulmonary artery saturation may further decrease left ventricle saturation due to an increase in venous admixture (Takala, 2007). Resolution may be achieved through either repositioning the drainage cannula towards the SVC (Hou et al., 2015; Falk et al., 2022) or modification of the cannulation strategy. A common solution is V-AV ECMO (blood is drained from the IVC and injected in the SVC as well as the femoral artery) or in the case of a failing right ventricle VV-A ECMO (drainage from both the IVC and SVC) (Blandino Ortiz et al., 2021; Wilson et al., 2022). The key to improve differential hypoxia is increased drainage from the SVC, as oxygen-rich blood from the IVC then enters the RA and attenuates differential hypoxia (Hou et al., 2015; Berger et al., 2023).

### Transfer and monitoring of gas exchange in VA ECMO

Lung function often deteriorates during VA ECMO, mainly due to increased LV filling pressures with subsequent congestion of the pulmonary vasculature (Pasero et al., 2014). The artificial lung may provide complete gas exchange for the patient, and the mechanical power necessary for lung ventilation is transferred to the membrane lung (Bachmann et al., 2020a). The extent of this transfer is directly linked to the amount of blood passing through the native lung (Bachmann et al., 2021). Monitoring of native lung function is difficult, but may be achieved with assessment of true oxygen uptake and CO_2_ removal through volumetric measurements (Berger et al., 2023). To fully wean and remove the ECMO circuit, the native lung must tolerate sufficient cardiac output and provide adequate gas exchange. Monitoring the native cardiopulmonary unit in terms of gas exchange and cardiac output may therefore guide the weaning process. While traditional measurement of cardiac output by thermodilution may fail (Bachmann et al., 2020b), measurements of gas exchange allow assessment of native cardiac output and lung function (Berger et al., 2023). However, depending on differential hypoxia and varying CO_2_ content between the arterial and venous compartments of the artificial and native circuits, gas exchange in the artificial circuit may provide only limited information about the native circuit (Berger et al., 2023).

### Deterioration of pulmonary function due to flow cessation

Experimental data suggests that flow cessation and the absence of pulmonary blood flow may lead to fibrosis, reduced pulmonary compliance and lung necrosis (Koul et al., 1991). It appears that several pathophysiological interactions exist which may significantly impact the native pulmonary function. Increased pulmonary edema due to increases in left ventricular filling pressures, structural changes due to inflammation and absence of pulsatile flow worsened by systemic inflammation may lead to long-term lung injury (Roumy et al., 2020). Strategies to improve lung function may include unloading of the left ventricle, prompt and adequate treatment of pneumonia and pre-weaning bronchoscopy (Luedi et al., 2018; Roumy et al., 2020), and optimized ventilation strategies (Rali et al., 2023).

## VV ECMO

### Impact on mechanical power applied to the native lung

Veno-venous ECMO has the potential benefit of reducing ventilator induced lung injury. In animal studies, near apneic ventilation has significantly decreased histological lung damage in a model of severe ARDS (Grandin et al., 2022). It is possible to transfer the work of CO_2_ removal from the native to the artificial lung, and native CO_2_ removal is inversely proportional to artificial CO_2_ removal (Berger et al., 2023). However, transitional changes in CO_2_ removal may readily be compensated by extensive CO_2_ storages in the body (Giosa et al., 2021). Reducing mechanical power may prove to be the goal in VV ECMO therapy, but optimal ventilator settings remain unknown. Although, in a model of iso-energetic ventilation, similar power application led to similar lung damage independently of the components of the mechanical power (Cressoni et al., 2016), ventilation strategies should aim to optimize and protect lung, heart and remote organ function.

### Interactions with right ventricular function

**FIGURE 1:**
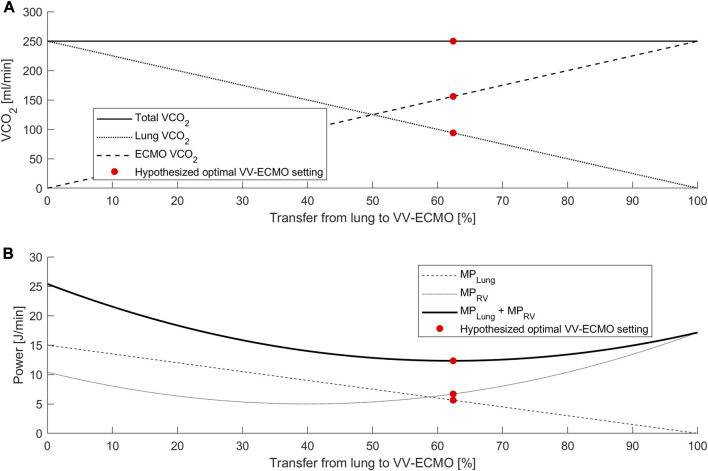
Theoretical analysis of proposed interaction between CO_2_ removal, mechanical power, and RV power. **(A)** Relationship between total VCO_2,_ VCO_2_ lung and VCO_2_ ECMO. An increase in the transfer from lung to VV ECMO reduces VCO_2_ lung in a linear relationship. **(B)** Mechanical power (MP) as a function of gas exchange transfer from the lung to the ECMO. While the mechanical power at the lung (MP_Lung_) is a linear function of lung VCO_2_, the RV power (MP_RV_) may form a u-shaped curve. A potential optimal setting (red point) refers to the minimum of the power sums and indicates the optimal points of gas exchange transfer.

A hallmark of acute respiratory distress syndrome is RV failure due to the burdens of increased afterload from hypoxic pulmonary vasoconstriction and mechanical ventilation (Zochios et al., 2017). Echocardiography may promptly identify patients with acute cor pulmonale and treatment strategies include initiation of VV ECMO (Vieillard-Baron et al., 2002; Petit et al., 2021). The findings of acute cor pulmonale in the setting of ARDS have led to the concept of RV protective ventilation (Vieillard-Baron et al., 2016; Petit et al., 2021), which may be facilitated by VV ECMO. In VV ECMO oxygenated blood is pumped into the pulmonary vasculature which ameliorates the effects of hypoxic pulmonary vasoconstriction (Holzgraefe et al., 2020; Zante et al., 2021). This effect depends on the prevailing degree of alveolar hypoxia, but substantial decreases in pulmonary vascular resistance and pulmonary artery pressures have been demonstrated through VV ECMO initiation (Reis Miranda et al., 2015; Holzgraefe et al., 2020). Furthermore, transfer of gas exchange to the artificial lung may reduce the need for mechanical ventilation with the potential to further reduce RV strain (Bunge et al., 2018). Right ventricular function may become an important factor in future strategies for VV ECMO initiation and weaning. Importantly, the level of support during ECMO therapy may also impact RV function: Total transfer of gas exchange and absolute lung rest may promote atelectasis with associated RV-strain while support at an inadequate level may risk exacerbation of lung injury caused by mechanical ventilation (Spinelli et al., 2021). Optimal settings should be chosen considering both RV strain and the mechanical power applied to the lung. Figure 1 provides a theoretical illustration of this concept.

### Venous admixture and arterial oxygen saturation

Arterial saturations are dependent on cardiac output, V/Q ratio in the lung, and venous oxygen content. In states of high shunt, as may be present in up to 50% of ARDS patients, low cardiac output states with low venous oxygen saturations may heavily impact oxygen saturation in the arterial system (Takala, 2007). In the setting of VV ECMO, the venous admixture is dependent on the ratio of ECMO flow to cardiac output (Schmidt et al., 2013) as well as the respective oxygen contents. In cases of high cardiac output and significant shunt, arterial saturations may decrease in a curvilinear function as the ratio of ECMO flow to cardiac output decreases (Zante et al., 2021). However, even if arterial oxygen saturation decreases with higher cardiac output, oxygen delivery will improve (Zante et al., 2021).

### Effect of respiratory quotient on end-expiratory lung volumes

Gas exchange from an artificial lung will affect the gas content in the pulmonary artery and thereby modify gas exchange ratios in the native lung. With inadequate oxygenation and CO_2_ removal mainly occurring through the artificial lung, the respiratory quotient (RQ = VCO_2_/VO_2_) of the native lung is reduced (Cipriani et al., 2020; Gattinoni et al., 2022). This impacts the alveolar gas composition, in particular the alveolar pO_2_ and oxygenation. Gattinoni and others demonstrated already in 1978 that depending on the RQ, higher fractions of inspired oxygen concentrations are necessary to produce an arterial saturation of 90%–94% (Gattinoni et al., 1978). A recently published theoretical analysis demonstrated that if CO_2_ removal is provided completely through the artificial lung and the RQ of the native lung reaches values <0.1, an FiO_2_ of 1.0 may be necessary to reach pO_2_ values of only 100 mmHg (Cipriani et al., 2020). Physicians operating ECMO or ECCO_2_R should be aware that inadvertent hypoxia may occur if the artificial lung is primarily used to remove CO_2_ without providing oxygenation. If the artificial lung also provides oxygenation, this effect is drastically diminished. The gas content in the pulmonary artery not only impacts the respiratory quotient, but also affects the nitrogen content of blood and alveoli. High oxygen fractions in the native lung will increase alveolar nitrogen washout over time, potentially leading to resorption atelectasis (Gattinoni, 2016). Although also demonstrated already in the 1970s (Kolobow et al., 1978), this effect has not been investigated in the setting of modern ECMO therapy for ARDS. Therefore, future physiological studies of severe ARDS should examine whether changes in sweep gas oxygen fraction may help to stabilize end-expiratory lung volumes.

## Discussion

Extracorporeal therapy has a major impact on cardiovascular and pulmonary physiology, and clinical management, especially in absence of data from large clinical studies, should rely on sound physiological concepts. Understanding these concepts may help clinicians adapt management strategies with the goal of improving daily clinical care and ultimately patient outcome.

In VA ECMO, both the cardiac and pulmonary physiology are severely altered: While the RV is inherently unloaded in VA ECMO, LV afterload is always increased, and LV function may deteriorate substantially if important measures such as adequate consideration to ECMO flow and principles of LV unloading are not considered. To increase maximum achievable ECMO flow and to allow further increase in oxygen delivery, factors enabling venous return must be understood. When operating at staccato flow, further increase in pump speed will only worsen vascular collapse as vessel walls, already with negative transmural pressure, are being pushed towards the ECMO cannula orifices. At this point, only improvement of factors promoting venous return can increase the potential ECMO flow. In VV ECMO, interactions revolve around the right ventricle as well as the pulmonary function. The concept of venous return holds true in VV ECMO, but as the drainage and return cannula may communicate through the venous system, increases in pump speed do not lead to vessel collapse but rather increase recirculation, without the potential to improve patient oxygenation. Effects of VV ECMO on right ventricular function, particularly afterload reduction (Reis Miranda et al., 2015; Holzgraefe et al., 2020; Petit et al., 2021) should be incorporated into treatment strategies, and changes in gas exchange and resulting arterial oxygen saturations depending on RQ, nitrogen content and venous admixture are important for daily clinical management (Ficial et al., 2021; Zante et al., 2021).

Future studies should concentrate on physiological questions. In VA ECMO, these questions should focus on maintaining LV and pulmonary function until organ recovery can be achieved. In VV ECMO the optimal device and ventilator settings remain unclear, but the strategy should consider gas composition, RV function, and minimized collateral damage to remote organs. Standardized monitoring strategies of cardiopulmonary function needs to be defined including new approaches such as integral gas exchange assessment and adapted thermodilution (Bachmann et al., 2020b; Bachmann et al., 2021; Berger et al., 2023).

Large clinical trials may help to answer questions regarding mortality or long-term patient outcome, but small physiological animal and patient studies, and computational physiological modeling, are necessary to fully understand the impact of extracorporeal therapy on the cardiopulmonary system, and to adapt and optimize the entry, maintenance and exit strategies of these devices and to identify the important research questions that need to be answered.

## References

[B1] Abrams D. MacLaren G. Lorusso R. Price S. Yannopoulos D. Vercaemst L. (2022). Extracorporeal cardiopulmonary resuscitation in adults: evidence and implications. Intensive Care Med. 48 (1), 1–15. 10.1007/s00134-021-06514-y 34505911 PMC8429884

[B2] Bachmann K. F. Haenggi M. Jakob S. M. Takala J. Gattinoni L. Berger D. (2020a). Gas exchange calculation may estimate changes in pulmonary blood flow during veno-arterial extracorporeal membrane oxygenation in a porcine model. Am. J. Physiol-Lung Cell Mol. Physiol. 318 (6), L1211–21. 10.1152/ajplung.00167.2019 32294391 PMC7276983

[B3] Bachmann K. F. Vasireddy R. Heinisch P. P. Jenni H. Vogt A. Berger D. (2021). Estimating cardiac output based on gas exchange during veno-arterial extracorporeal membrane oxygenation in a simulation study using paediatric oxygenators. Sci. Rep. 11 (1), 11528. 10.1038/s41598-021-90747-w 34075067 PMC8169686

[B4] Bachmann K. F. Zwicker L. Nettelbeck K. Casoni D. Heinisch P. P. Jenni H. (2020b). Assessment of right heart function during extracorporeal therapy by modified thermodilution in a porcine model. Anesthesiology 133 (4), 879–891. 10.1097/ALN.0000000000003443 32657798

[B5] Berger D. Moller P. W. Takala J. (2019). Reply to “Is the Guytonian framework justified in explaining heart lung interactions?” and “Venous return, mean systemic pressure and getting the right answer for the wrong reason”. Ann. Transl. Med. 7 (8), 186. 10.21037/atm.2019.04.50 31169266 PMC6526252

[B6] Berger D. Moller P. W. Takala J. (2016b). Reply to “letter to the editor: why persist in the fallacy that mean systemic pressure drives venous return?”. Am. J. Physiol-Heart Circ. Physiol. 311 (5), H1336–7. 10.1152/ajpheart.00622.2016 27802975

[B7] Berger D. Moller P. W. Weber A. Bloch A. Bloechlinger S. Haenggi M. (2016a). Effect of PEEP, blood volume, and inspiratory hold maneuvers on venous return. Am. J. Physiol. Heart Circ. Physiol. 311 (3), H794–H806. 10.1152/ajpheart.00931.2015 27422991

[B8] Berger D. C. Zwicker L. Nettelbeck K. Casoni D. Heinisch P. P. Jenni H. (2023). Integral assessment of gas exchange during veno-arterial ECMO: accuracy and precision of a modified fick principle in a porcine model. Am. J. Physiol. Lung Cell Mol. Physiol. 324 (2), L102–L113. 10.1152/ajplung.00045.2022 36511508 PMC9870575

[B9] Blandino Ortiz A. Belliato M. Broman L. M. Lheureux O. Malfertheiner M. V. Xini A. (2021). Early findings after implementation of veno-arteriovenous ECMO: A multicenter European experience. Membranes 11 (2), 81. 10.3390/membranes11020081 33499236 PMC7912524

[B10] Bunge J. J. H. Caliskan K. Gommers D. Reis Miranda D. (2018). Right ventricular dysfunction during acute respiratory distress syndrome and veno-venous extracorporeal membrane oxygenation. J. Thorac. Dis. 10 (5), S674–82. 10.21037/jtd.2017.10.75 29732186 PMC5911554

[B11] Burkhoff D. Sayer G. Doshi D. Uriel N. (2015). Hemodynamics of mechanical circulatory support. J. Am. Coll. Cardiol. 66 (23), 2663–2674. 10.1016/j.jacc.2015.10.017 26670067

[B12] Cipriani E. Langer T. Bottino N. Brusatori S. Carlesso E. Colombo S. M. (2020). Key role of respiratory quotient to reduce the occurrence of hypoxemia during extracorporeal gas exchange: A theoretical analysis. Crit. Care Med. 48 (12), e1327–e1331. 10.1097/CCM.0000000000004619 33031149

[B13] Combes A. Brodie D. Aissaoui N. Bein T. Capellier G. Dalton H. J. (2022). Extracorporeal carbon dioxide removal for acute respiratory failure: a review of potential indications, clinical practice and open research questions. Intensive Care Med. 48 (10), 1308–1321. 10.1007/s00134-022-06796-w 35943569

[B14] Cressoni M. Gotti M. Chiurazzi C. Massari D. Algieri I. Amini M. (2016). Mechanical power and development of ventilator-induced lung injury. Anesthesiology 124 (5), 1100–1108. 10.1097/ALN.0000000000001056 26872367

[B15] Donker D. W. Brodie D. Henriques J. P. S. Broomé M. (2019). Left ventricular unloading during veno-arterial ECMO: A simulation study. ASAIO J. 65 (1), 11–20. 10.1097/MAT.0000000000000755 29517515 PMC6325768

[B16] Donker D. W. Burkhoff D. Mack M. J. (2022). ECMO: we need to vent about the need to vent. J. Am. Coll. Cardiol. 79 (13), 1251–1253. 10.1016/j.jacc.2022.01.034 35361347

[B17] Donker D. W. Sallisalmi M. Broomé M. (2021). Right–left ventricular interaction in left-sided heart failure with and without venoarterial extracorporeal membrane oxygenation support—a simulation study. ASAIO J. 67 (3), 297–305. 10.1097/MAT.0000000000001242 33627604 PMC7908866

[B18] Falk L. Hultman J. Broman L. M. (2022). Differential hypoxemia and the clinical significance of venous drainage position during extracorporeal membrane oxygenation. Perfusion 38, 818–825. 10.1177/02676591221090667 35543368

[B19] Ficial B. Vasques F. Zhang J. Whebell S. Slattery M. Lamas T. (2021). Physiological basis of extracorporeal membrane oxygenation and extracorporeal carbon dioxide removal in respiratory failure. Membranes 11 (3), 225. 10.3390/membranes11030225 33810130 PMC8004966

[B20] Gattinoni L. Coppola S. Camporota L. (2022). Physiology of extracorporeal CO2 removal. Intensive Care Med. 48 (10), 1322–1325. 10.1007/s00134-022-06827-6 36006451 PMC9468086

[B21] Gattinoni L. Kolobow T. Tomlinson T. White D. Pierce J. (1978). Control of intermittent positive pressure breathing (IPPB) by extracorporeal removal of carbon dioxide. Br. J. Anaesth. 50 (8), 753–758. 10.1093/bja/50.8.753 354668

[B22] Gattinoni L. (2016). Ultra-protective ventilation and hypoxemia. Crit. Care Lond Engl. 20 (1), 130. 10.1186/s13054-016-1310-9 PMC4865006 27170273

[B23] Giosa L. Busana M. Bonifazi M. Romitti F. Vassalli F. Pasticci I. (2021). Mobilizing carbon dioxide stores. An experimental study. Am. J. Respir. Crit. Care Med. 203 (3), 318–327. 10.1164/rccm.202005-1687OC 32813989

[B24] Grandin E. W. Nunez J. I. Willar B. Kennedy K. Rycus P. Tonna J. E. (2022). Mechanical left ventricular unloading in patients undergoing venoarterial extracorporeal membrane oxygenation. J. Am. Coll. Cardiol. 79 (13), 1239–1250. 10.1016/j.jacc.2022.01.032 35361346 PMC9187498

[B25] Holzgraefe B. Larsson A. Eksborg S. Kalzén H. (2020). Does extracorporeal membrane oxygenation attenuate hypoxic pulmonary vasoconstriction in a porcine model of global alveolar hypoxia? Acta Anaesthesiol. Scand. 64 (7), 992–1001. 10.1111/aas.13588 32236954

[B26] Hou X. Yang X. Du Z. Xing J. Li H. Jiang C. (2015). Superior vena cava drainage improves upper body oxygenation during veno-arterial extracorporeal membrane oxygenation in sheep. Crit. Care 19 (1), 68. 10.1186/s13054-015-0791-2 25887895 PMC4352275

[B27] Jayaraman A. L. Cormican D. Shah P. Ramakrishna H. (2017). Cannulation strategies in adult veno-arterial and veno-venous extracorporeal membrane oxygenation: techniques, limitations, and special considerations. Ann. Card. Anaesth. 20 (Suppl 1), S11–S18. 10.4103/0971-9784.197791 28074818 PMC5299823

[B28] Kolobow T. Gattinoni L. Tomlinson T. Pierce J. E. (1978). An alternative to breathing. J. Thorac. Cardiovasc Surg. 75 (2), 261–266. 10.1016/s0022-5223(19)41297-x 625133

[B29] Koul B. Willen H. Sjöberg T. Wetterberg T. Kugelberg J. Steen S. (1991). Pulmonary sequelae of prolonged total venoarterial bypass: evaluation with a new experimental model. Ann. Thorac. Surg. 51 (5), 794–799. 10.1016/0003-4975(91)90128-d 2025083

[B30] Kowalewski M. Malvindi P. G. Zieliński K. Martucci G. Słomka A. Suwalski P. (2020). Left ventricle unloading with veno-arterial extracorporeal membrane oxygenation for cardiogenic shock. Systematic review and meta-analysis. J. Clin. Med. 9 (4), 1039. 10.3390/jcm9041039 32272721 PMC7230555

[B31] Luedi M. Friess J. O. Erdoes G. (2018). Veno-arterial ECMO weaning failure in the operating room: have you considered preweaning bronchoscopy? Artif. Organs 42 (12), 1234–1235. 10.1111/aor.13272 30027654

[B32] Magder S. (2016). Volume and its relationship to cardiac output and venous return. Crit. Care 20 (1), 271. 10.1186/s13054-016-1438-7 27613307 PMC5018186

[B33] Moller P. W. Hana A. Heinisch P. P. Liu S. Djafarzadeh S. Haenggi M. (2019). The effects of vasoconstriction and volume expansion on veno-arterial ECMO flow. Shock Augusta Ga 51 (5), 650–658. 10.1097/SHK.0000000000001197 29877960

[B34] Moller P. W. Winkler B. Hurni S. Heinisch P. P. Bloch A. Sondergaard S. (2017). Right atrial pressure and venous return during cardiopulmonary bypass. Am. J. Physiol. Heart Circ. Physiol. 313 (2), H408–20. 10.1152/ajpheart.00081.2017 28550170

[B35] Pasero D. Persico P. Tenaglia T. Ranieri V. M. (2014). “Respiratory monitoring during VA ECMO,” in ECMO-extracorporeal life support in adults. Editors Sangalli F. Patroniti N. Pesenti A. (Milano: Springer Milan), 383–388. 10.1007/978-88-470-5427-1_33

[B36] Petit M. Jullien E. Vieillard-Baron A. (2021). Right ventricular function in acute respiratory distress syndrome: impact on outcome, respiratory strategy and use of veno-venous extracorporeal membrane oxygenation. Front. Physiol. 12, 797252. 10.3389/fphys.2021.797252 35095561 PMC8795709

[B37] Rali A. S. Tran L. E. Auvil B. Xu M. Huang S. Labrada L. (2023). Modifiable mechanical ventilation targets are associated with improved survival in ventilated VA-ECLS patients. JACC Heart Fail 11, 961–968. 10.1016/j.jchf.2023.03.023 37178085 PMC10171237

[B38] Reis Miranda D. van Thiel R. Brodie D. Bakker J. (2015). Right ventricular unloading after initiation of venovenous extracorporeal membrane oxygenation. Am. J. Respir. Crit. Care Med. 191 (3), 346–348. 10.1164/rccm.201408-1404LE 25635492

[B39] Roumy A. Liaudet L. Rusca M. Marcucci C. Kirsch M. (2020). Pulmonary complications associated with veno-arterial extra-corporeal membrane oxygenation: a comprehensive review. Crit. Care Lond Engl. 24 (1), 212. 10.1186/s13054-020-02937-z PMC7216520 32393326

[B40] Schmidt M. Tachon G. Devilliers C. Muller G. Hekimian G. Bréchot N. (2013). Blood oxygenation and decarboxylation determinants during venovenous ECMO for respiratory failure in adults. Intensive Care Med. 39 (5), 838–846. 10.1007/s00134-012-2785-8 23291732

[B41] Schrage B. Becher P. M. Bernhardt A. Bezerra H. Blankenberg S. Brunner S. (2020). Left ventricular unloading is associated with lower mortality in patients with cardiogenic shock treated with venoarterial extracorporeal membrane oxygenation: results from an international, multicenter cohort study. Circulation 142 (22), 2095–2106. 10.1161/CIRCULATIONAHA.120.048792 33032450 PMC7688081

[B42] Schrage B. Sundermeyer J. Blankenberg S. Colson P. Eckner D. Eden M. (2023). Timing of active left ventricular unloading in patients on venoarterial extracorporeal membrane oxygenation therapy. JACC Heart Fail 11 (3), 321–330. 10.1016/j.jchf.2022.11.005 36724180

[B43] Spinelli E. Colussi G. Dal Santo G. Scotti E. Marongiu I. Garbelli E. (2021). Atelectasis, shunt, and worsening oxygenation following reduction of respiratory rate in healthy pigs undergoing ECMO: an experimental lung imaging study. Front. Physiol. 12, 663313. 10.3389/fphys.2021.663313 33897471 PMC8063114

[B44] Takala J. (2007). Hypoxemia due to increased venous admixture: influence of cardiac output on oxygenation. Intensive Care Med. 33 (5), 908–911. 10.1007/s00134-007-0546-x 17342520

[B45] Truby L. K. Takeda K. Mauro C. Yuzefpolskaya M. Garan A. R. Kirtane A. J. (2017). Incidence and implications of left ventricular distention during venoarterial extracorporeal membrane oxygenation support. ASAIO J. Am. Soc. Artif. Intern Organs 63 (3), 257–265. 10.1097/MAT.0000000000000553 28422817

[B46] Vieillard-Baron A. Matthay M. Teboul J. L. Bein T. Schultz M. Magder S. (2016). Experts’ opinion on management of hemodynamics in ARDS patients: focus on the effects of mechanical ventilation. Intensive Care Med. 42 (5), 739–749. 10.1007/s00134-016-4326-3 27038480

[B47] Vieillard-Baron A. Prin S. Chergui K. Dubourg O. Jardin F. (2002). Echo-Doppler demonstration of acute cor pulmonale at the bedside in the medical intensive care unit. Am. J. Respir. Crit. Care Med. 166 (10), 1310–1319. 10.1164/rccm.200202-146CC 12421740

[B48] Vyas A. Bishop M. A. (2022). Extracorporeal membrane oxygenation in adults. Treasure Island (FL): StatPearls Publishing. 35015451

[B49] Werner-Moller P. Berger D. Takala J. (2020). Letter to the editor: venous return and the physical connection between distribution of segmental pressures and volumes. Am. J. Physiol. Heart Circ. Physiol. 318 (1), H203-H204–4. 10.1152/ajpheart.00698.2019 31910359

[B50] Werner-Moller P. Heinisch P. P. Hana A. Bachmann K. F. Sondergaard S. Jakob S. M. (2022). Experimental validation of a mean systemic pressure analog against zero-flow measurements in porcine VA-ECMO. J. Appl. Physiol. Bethesda Md 1985 132 (3), 726–736. 10.1152/japplphysiol.00804.2021 35085032

[B51] Wilson J. Fisher R. Caetano F. Soliman-Aboumarie H. Patel B. Ledot S. (2022). Managing Harlequin Syndrome in VA-ECMO - do not forget the right ventricle. Perfusion 37 (5), 526–529. 10.1177/02676591211020895 34053349

[B52] Zante B. Berger D. C. Schefold J. C. Bachmann K. F. (2021). Dissociation of arterial oxygen saturation and oxygen delivery in VV-ECMO: the trend is your friend. J. Cardiothorac. Vasc. Anesth. 35 (3), 962–963. 10.1053/j.jvca.2020.06.084 32739087 PMC7332908

[B53] Zochios V. Parhar K. Tunnicliffe W. Roscoe A. Gao F. (2017). The right ventricle in ARDS. Chest 152 (1), 181–193. 10.1016/j.chest.2017.02.019 28267435

